# Seroprevalence and risk factors for brucellosis in small ruminant flocks in Karnataka in the Southern Province of India

**DOI:** 10.14202/vetworld.2021.2855-2862

**Published:** 2021-11-09

**Authors:** Krithiga Natesan, Triveni Kalleshamurthy, Mangadevi Nookala, Chaitra Yadav, Nagalingam Mohandoss, Somy Skariah, Swati Sahay, Bibek Ranjan Shome, Obli Rajendran Vinodh Kumar, Habibur Rahman, Rajeswari Shome

**Affiliations:** 1 Department of Bacteriology, Indian Council for Agricultural Research-National Institute of Veterinary Epidemiology and Disease Informatics, Bengaluru, Karnataka, India; 2 Division of Epidemiology, Indian Council for Agricultural Research-Indian Veterinary Research Institute, Bareilly, Uttar Pradesh, India; 3 International Livestock Research Institute, New Delhi, India

**Keywords:** brucellosis, goats, India, risk factors, sheep, seroprevalence

## Abstract

**Background and Aim:**

Brucellosis is a zoonotic disease of high economic and public health importance in large and small ruminant populations worldwide. A cross-sectional study was conducted to determine the seroprevalence and risk factors of brucellosis in small ruminants in organized farms in the southern region of India.

**Materials and Methods:**

Farms exclusively rearing sheep and goats were selected based on the number of animals (small, medium, or large) and the location of the farm (urban, periurban, or rural). A total of 1499 serum samples; 1001 from sheeps and 498 from goats were sourced from six sheep and four goat farms and tested using Rose Bengal Plate and indirect Enzyme-Linked Immunosorbent Assay tests.

**Results:**

The apparent prevalence of brucellosis was higher in sheep (8.29%, 95% CI 6.7-10.1) than goats (5.82%, 95% CI 4.0-8.2). The true adjusted population level seroprevalence was also higher in sheep, at 7.7% (95% CI 6.0-9.6) than in goats, at 5.1% (95% CI 3.2-7.6). According to bivariate categorical analysis, six highly significant (p<0.001) animal- and farm-level risk factors for sheep were age, breed, number of lambings, history of abortion, rural farms, and presence of dogs on the farm. In goats, five significant risk factors were found: History of abortion, separate sheds, dogs on the farm, weekly veterinary consultation, and lack of brucellosis awareness. In a logistic regression model, abortion (OR _adjusted_ 10.8, 95% CI 1.2-96.12), rural farms (OR _adjusted_ 8.5, 95% CI 3.6-20.0), and absence of separate sheds on the farms (OR 1.9, 95% CI 1.1-3.5) were found to be significant risk factors for ovine brucellosis.

**Conclusion:**

The use of complementary measures to tackle the multiple animal- and farm-level risk factors may help to reduce the disease burden in the absence of a vaccination policy for small ruminants in India.

## Introduction

Brucellosis is an infectious disease caused by bacteria of the genus *Brucella*, and is characterized by abortion and infertility in several mammalian species. It is considered to be one of the most important zoonoses worldwide [1]. The disease is endemic in Latin America, Asia, some Mediterranean regions, and Africa, including India [2]. In small ruminants, the disease is primarily caused by *Brucella melitensis*, although *Brucella ovis* is also an important cause of orchitis and epididymitis in rams, and occasionally in ewes [3]. A few cases of *Brucella abortus* and *Brucella suis* have been reported in small ruminants [4]. Transmission of brucellosis occurs primarily by the ingestion of contaminated feed and water, sexual contact, or direct contact with infected placental or uterine discharges [5]. Several risk factors are associated with its transmission [6] and these risk factors may depend on various animal and environment related factors, such as the species, geographical area, and rearing practices [7].

Brucellosis a major cause of economic losses in terms of abortion, infertility, decreased milk production, culling of infected animals, and treatment costs [6]. Brucellosis has serious negative socioeconomic impacts on people, due to the loss of work and income caused by the illness [8,9]. Brucellosis is endemic to India, and economic impact estimates for livestock are annual median losses of US $ 3.4 billion and in humans, US $ 6.39 and 2.67 million among adults and offspring were recorded, respectively [10,11].

Brucellosis can be controlled in many ways, including periodical surveillance to locate hot spots, reducing the transmitting risk factors, isolation of seropositive animals, and compulsory vaccination [2]. The seroprevalence of brucellosis in sheep and goat has been reported to be 7.9% and 2.2%, respectively, at the country level [12], and recently a few regional reports have emerged [13-16]. A higher prevalence of brucellosis (19.20%) was reported in unorganized sector/field conditions than in the organized sector (8.10%), as assessed using indirect Enzyme-Linked Immunosorbent Assay (iELISA) [16], contrary to another report about proper biosecurity and routine screening of animals for brucellosis [17].

The study aimed to asses seroprevalence of brucellosis and to identify the brucellosis risk factors associated with small ruminant brucellosis in the organized rearing flocks. In the absence of a vaccination policy for small ruminants in the country, the identification of risk factors will aid in designing practices to curtail disease transmission in small ruminants reared exclusively in the organized sector.

## Materials and Methods

### Ethical approval

The study was approved by the Institutional Animal Ethics Committee, ICAR- NIVEDI, Bengaluru, India under the DBT Network Project Brucellosis/IFD/SAN/3142/2012-13 dated 27-09-2012.

### Study area, period, design, and data collection

Karnataka state is in the Deccan Plateau of India, it is bordered by six states and the Arabian Sea to the west, and about 54% of the total geographical area is drought-prone. Small ruminants have higher importance than agricultural crops, as they can be sustained with minimal input, and are a viable source of livelihood for more than 50% of farmers in this drought-prone area [18].

A cross-sectional study was conducted from January 2015 to January 2017 to investigate brucellosis in organized small ruminant flocks. In the first stage, we contacted the farmers personally and appraised them of the purpose of the study, based on the databases provided by the Department of Animal Husbandry, Government of Karnataka, Republic of India. While selecting the flocks, farmers maintaining only sheep or goats were included in the study, and the farmers rearing both sheep and goats or sheep or goats with cattle or buffalo were excluded from the study to estimate the exclusive species-wise prevalence of brucellosis. Ten farms of this type were chosen based on their location with respect to a city (periurban or rural), and the farmers’ consent to complete a questionnaire survey. The final farms included six flocks of sheep, two periurban and four rural, and four flocks of goats, two each periurban and rural. Responses about farm practices and animal details were collected from animal handlers and farm managers using a closed-ended, pretested questionnaire. For each farm, the sample size was estimated using the sampling book package in R (http://www.R-project.org/) with 5-40% seroprevalence, considering the reported seroprevalence in the state at a 0.02 precision, 95% confidence level.

The Epidata responses include species (sheep, goat), sex (male/female), sheep breeds (Bannur, Bellary, Deccani, Hassan, Kenguri, or Indigenous), goat breeds (Barbari, Beetal, Boar, Jamanapari, Sirohi, or Indigenous), age (stratified into less than 8 months and 8 months or above), number of lambings or kiddings (0-2, 3-5, or 6-8), and reproductive history of the animal (abortion, retention of placenta, hygroma, or orchitis). The flocks were classified as periurban or rural. Periurban was defined as occurring in the rural-urban transition zone, while rural was defined as the area located outside the town and city premises. The flocks were classified as small (50-75 adult animals), medium (100-200 adult animals), or large (more than 200 adult animals). The rearing methods were categorized as extensive (animals left free and maintained only by grazing with no provision of concentrates) or semi-intensive (animals left free during the day for grazing, kept in-house after grazing, and provided with concentrate). Other variables included the mode of procurement of animals (own raised and procured from livestock fairs) and the presence or absence of separate sheds in the farm for young, sick, or pregnant animals. The farm practices such as the method of disposal of aborted material or fetuses in open fields or water bodies, the role of dogs in disease transmission, the frequency of veterinarian visits (weekly or monthly), and brucellosis awareness were collected and correlated with the disease occurrence.

### Sample collection and serological screening

Approximately 3-5 mL of blood was collected from the jugular vein using vacutainers without ethylenediaminetetraaceticacid (Becton Dickinson, Franklin Lakes, NJ, USA). Serum was separated from clotted blood after 4-6 h by centrifugation at 5000× *g* for 5-10 min to obtain clear serum. The samples were tested using Rose Bengal plate tests (RBPT) by mixing 30 mL of serum and antigen (IAH and VB, Hebbal, Bengaluru, India) on a glass slide. A definite clumping/agglutination within 3 min was considered to be a positive result, while no clumping/agglutination was taken to be negative [19]. The previously standardized ELISA was used for testing the serum samples. Serum samples were tested using iELISA with smooth lipopolysaccharide antigen and rabbit antisheep IgG-HRP conjugate (Sigma-Aldrich, St. Louis, MO, USA), and samples with a positivity value below 54% were taken as negative, and those with more than 54% were considered as positive [13].

### Statistical analysis

Information from the questionnaire was digitized into a Microsoft Excel spreadsheet (Microsoft, Redmond, WA, USA) and serological test scores were assigned as seronegative=0 or seropositive=1. The continuous variables such as the number of times animals had given birth, number of animals, and age were converted to categorical data, and the data were imported into R version 3.1.1. Apparent prevalence (AP) and true prevalence were calculated at 95% confidence with sensitivity of 96% and specificity of 99% using iELISA (https://epitools.ausvet.com.au). Bivariate categorical analysis was performed with seropositivity as the dependent variable, and the factors that were likely to predict the outcome as independent variables. The degree of association between the potential risk factors and seroprevalence was calculated using the odds ratio (OR), and confounders were identified using strata specific OR. A multivariable logistic regression model was built using a forward conditional approach with the potential risk factors, with p<0.2 recognized from the bivariate analysis. The final model fit was assessed using the Hosmer-Lemeshow test, and in the final model, only those factors significant at p≤0.05 were retained.

## Results and Discussion

Small ruminants (sheep and goats) are the backbone of the Indian agrarian economy [18-21]. Of 1499 serum samples, 1001 from sheeps, and 498 from goats were collected from six organized sheep farms and four organized goat farms, and the samples found to be positive using both RBPT and iELISA were used for further analysis. At the animal level, we recorded a higher AP in sheep (8.29%, 95% CI 6.7-10.1) than in goats (5.82%, 95% CI, 4.0-8.2) (Table-1). In the study, more sheep samples were analyzed, because Karnataka state has 9.57 ­million sheep and only 3.5 million goats. From the same region, the brucellosis seroprevalence in goat flocks with a history of abortions was 11.30% [22], 8.57% in organized sheep farms, and 2.73% in goat farms [23]. In surveys conducted in unorganized settings, the seroprevalence was 7.9% in sheep and 2.20% in goats according to RBPT and ELISA [12], and 8.7% in sheep and 5.8% in goats according to iELISA were reported [13]. Based on the above mentioned studies and the present results, the overall brucellosis seroprevalence was 8%-9% in sheep and 2%-5% in goats in both organized and unorganized rearing systems, indicating the conserved and persistent presence of the disease in the small ruminant population.

**Table-1:** Brucellosis seroprevalence in sheep and goat flocks of Karnataka, India.

Place of collection	Latitude	Longitude	Total number of animals in the farm	Total samples collected	Total positives	AP % (95% CI)	TP % (95% CI)
Sheep farms							
Bellary-urban	15.1394° N	76.9214° E	1823	374	42	11.23 (8.42-14.83)	10.77 (7.81-14.56)
Mysuru- urban	12.2958° N	76.6394° E	879	197	07	3.55 (1.73-7.15)	2.69 (0.77-6.48)
Bidar-rural	17.9149° N	77.5046° E	120	32	0	0 (0-0.1072)	NC
Hassan-rural	13.0068° N	76.0996° E	967	257	34	13.23 (9.62-17.92)	12.87 (9.08-17.81)
Mandya-rural	12.5222° N	76.9009° E	319	127	0	0 (0-2.94)	NC
Raichur-rural	16.2120° N	77.3439° E	65	14	0	0 (0-2.15)	NC
Total			4173	1001	83	8.29 (6.74-10.16)	7.68 (6.04-9.65)
Goat farms							
Hassan-periurban	13.0068° N	76.0996° E	125	50	01	2.0 (0.35-10.5)	1.05 (0-10.0)
Kolar- periurban	13.1362° N	78.1291° E	45	15	03	20.0 (7.0-45.0)	20.0 (6. 4-47.0)
Mandya-rural	12.5222° N	76.9009° E	868	252	14	5.56 (3.34-9.11)	4.8 (2.46-8.53)
Mysuru-rural	12.2958° N	76.6394° E	541	181	11	6.08 (3.43-10.55)	5.34 (2.55-10.1)
Total			1579	498	29	5.82 (4.08-8.24)	5.08 (3.25-7.62)

CI=Confidence interval, NC=Not calculated, AP: Apparent prevalence, TP: True prevalence. iELISA sensitivity is 96% and specificity is 99

At the animal level, *Brucella* seropositivity was significantly higher in the 8 months and above age group of animals in both species of livestock (OR 2.49, 95% CI 0.8-7.2), possibly because the disease affects actively reproducing animals [24]. The highest susceptibility to brucellosis in sheep was reported in the 3 years of age and above group from Central and Northeast Ethiopia [25], and in the more than 1 year of age group in goats from Sudan [26]. Animals with three to four parities showed statistically significantly (p<0.001) higher odds for brucellosis in both sheep and goats, in keeping with the previous findings that brucellosis infection is always recorded in sexually mature adults [27] and pregnant animals [5] (Table-2).

**Table-2:** Animal level risk factors for brucellosis seropositivity in sheep.

Criteria	Risk factors	Number of samples (1001)	Seropositives (83)	OR and inverse OR^ѱ^ (95% CI)	p-value
Age	Below 8 months	358 (35.76)	15 (18.07)	0.40 (0.22-0.70)	0.001***
	Above 8 months	643 (64.24)	68 (81.93)	2.52 (1.42-4.48)	
Sex	Female (ewe)	953 (95.20)	78 (93.98)	0.79 (0.30-2.03)	0.618
	Male (ram)	48 (4.80)	5 (6.02)	1.27 (0.49-3.29)	
Breed	Bannur	285 (28.47)	5 (6.02)	0.16 (0.06-0.40)	0.001***
	Bellary	374 (37.36)	42 (50.60)	1.72 (1.20-2.70)	0.02*
	Deccani	32 (3.20)	0 (0.00)	NC	0.098*
	Hassan	257 (25.67)	34 (40.96)	2.01 (1.27-3.18)	0.01**
	Kenguri	14 (1.40)	0 (0.00)	NC	0.278
	Indigenous	39 (3.90)	2 (2.41)	0.61 (0.14-2.57)	0.495
Number of Lambings	0-2	376 (37.56)	15 (18.07)	0.37 (0.21-0.65)	0.001***
	3-4	322 (32.17)	44 (53.01)	2.38 (1.51-3.73)	0.001***
	5-8	303 (30.27)	24 (28.92)	0.94 (0.57-1.53)	0.796
History	Abortion	58 (5.79)	29 (34.94)	8.73 (5.17-14.74)	0.000***
	Hygroma	6 (0.60)	0 (0.00)	NC	0.024*
	Orchitis	13 (1.30)	1 (1.20)	0.93 (0.12-7.17)	0.942
	No symptoms	924 (92.31)	53 (63.86)	0.15 (0.09-0.24)	0.000***

CI=Confidence interval, NC=Not calculated.

*** p<0.001,

** p<0.01,

* p<0.1, ^ѱ^The reference is the other paired group

The odds of brucellosis were greater for rams than for ewes (OR 1.27, 95% CI 0.4-3.2), while it was higher for does than bucks (OR 1.19, 95% CI 0.3-4.0) (Table-3). The high brucellosis seroprevalence in rams was probably due to the exposure of males to many females during mating [28], and in the absence of artificial insemination for small ruminants in the country, brucellosis infected male animals may pose a greater risk of disease transmission through natural breeding on the farms. A significantly (p<0.001) higher rate of brucellosis seropositive was found in Hassan and Bellary breeds of sheep, possibly due to the large number of samples collected from popular meat breeds of the region. In case of goats, the Barbari breed exhibited higher odds (OR 2.35, 95% CI 1.0-5.2), whereas no association was observed in goat breeds by Wukari [28]. As well as management factors, we looked at breed predisposition to brucellosis among the sampled animals (Table-4). Brucellosis seropositivity was significantly higher in goats (OR 52.12, 95% CI 15.3-176.7) than in sheep (OR 8.73, 95% CI 5.1-14.7) in animals with a clinical history of abortion, as has been found by several previous studies [25,29-32].

**Table-3:** Farm level risk factors for brucellosis seropositivity in sheep.

Criteria	Risk factors	Number of samples (1001)	Seropositives (83)	OR and inverse OR^ѱ^ (95% CI)	p-value
Location of the farm	Rural	631 (63.04)	76 (91.57)	6.37 (2.90-13.95)	0.001***
	Peri urban	370 (36.96)	7 (8.43)	0.16 (0.07-0.34)	
Number of animals in the farm	Small farm	29 (2.90)	3 (3.61)	1.26 (0.37-4.21)	0.710
	Medium Farm	191 (19.08)	22 (26.51)	1.53 (0.91-2.55)	0.102
	Large Farm	780 (77.92)	58 (69.88)	0.66 (0.40-1.07)	0.092*
Method of rearing	Extensive	126 (12.59)	9 (10.84)	0.84 (0.41-1.73)	0.644
	Semi intensive	875 (87.41)	74 (89.16)	1.18 (0.58-2.42)	
Mode of procurement of animals	Own raised	799 (79.82)	58 (69.88)	0.59 (0.36-0.96)	0.03*
	Livestock fair	202 (20.17)	25 (30.12)	1.70 (1.04-2.79)	
Disposal of aborted materials/foetus	Open discard	425 (42.46)	25 (30.12)	0.58 (0.36-0.95)	0.03*
	Disposed in water bodies	576 (57.54)	58 (69.88)	1.71 (1.05-2.78)	
Separate shed	Yes	807 (80.62)	62 (74.70)	0.71 (0.42-1.19)	0.194
	No	194 (19.38)	21 (25.30)	1.41 (0.84-2.37)	
Dogs in the farm	Yes	460 (45.95)	59 (71.08)	2.89 (1.77-4.72)	0.001***
	No	541 (54.05)	24 (28.92)	0.35 (0.21-0.56)	
Frequency of Veterinary services obtained	Weekly	220 (21.98)	25 (30.12)	1.59 (0.97-2.62)	0.06*
	Monthly	781 (78.02)	58 (69.88)	0.65 (0.40-1.07)	
Brucellosis Awareness	Yes	623 (62.24)	53 (63.86)	1.07 (0.67-1.72)	0.77
	No	378 (37.76)	30 (36.14)	0.93 (0.59-1.49)	

CI=Confidence interval, OR: Odds ratio.

*** p<0.001,

**p<0.01,

* P*<*0.1, ^ѱ^the reference is the other paired group

**Table-4:** Animal level risk factors for brucellosis seropositivity in goats.

Criteria	Risk factors	Number of samples (498)	Seropositives (29)	OR and inverse OR^ѱ^ (95% CI)	p-value
Age	Below 8 months	142 (28.51)	4 (13.79)	0.40 (0.14-1.17)	0.08*
	Above 8 months	356 (71.49)	25 (86.21)	2.49 (0.85-7.29)	
Sex	Female (doe)	438 (87.95)	26 (89.66)	1.19 (0.35-4.04)	0.784
	Male (buck)	60 (12.05)	3 (10.34)	0.84 (0.25-2.87)	
Breed	Barbari	91 (18.27)	10 (34.48)	2.35 (1.05-5.23)	0.031*
	Beetal	12 (2.41)	1 (3.45)	1.44 (0.18-11.52)	0.726
	Boar	49 (9.84)	5 (17.24)	1.91 (0.70-5.23)	0.201
	Jamanapari	147 (29.52)	9 (31.03)	1.07 (0.47-2.42)	0.862
	Sirohi	143 (28.71)	1 (3.45)	0.09 (0.01-0.66)	0.003**
	Indigenous	56 (11.24)	3 (10.34)	0.90 (0.27-3.11)	0.881
Number of Kiddings	0-2	159 (31.93)	6 (20.69)	0.56 (0.22-1.39)	0.205
	3-4	193 (38.76)	13 (44.83)	1. 28 (0.60-2.73)	0.515
	5-8	146 (29.32)	10 (34.48)	1.27 (0.58-2.80)	0.553
History	Abortion	71 (14.26)	26 (89.66)	52.12 (15.37-176.76)	0.000***
	Hygroma	12 (2.41)	0 (0.0)	NC	0.398
	Orchitis	11 (2.21)	1 (3.45)	1.58 (0.20-12.69)	0.0663*
	No symptoms	404 (81.12)	2 (6.90)	0.02 (0.00-0.07)	0.000***

CI=Confidence interval, NC=Not calculated, OR=Odds ratio.

*** p<0.001,

** p<0.01,

* p<0.1, ^ѱ^the reference is the other paired group

At the farm level, significantly higher odds (p<0.001) (OR 6.37, 95% CI 2.9-13.9) of brucellosis seropositivity were observed in rural sheep flocks and in semi-intensive sheep rearing systems. The increased disease prevalence in semi-intensive systems was probably due to out-grazing with different herds, and shedding of the disease in the pens on return from pasture grazing and mating [28]. The opposite pattern was observed for goats, in which the odds were higher in periurban flocks and in extensive rearing systems of OR 3.72, CI 95% 1.0-13.6 (Table-5). The reason could be higher stocking density and close contact between animals in infected environments in the urban and periurban farms [8], or it could be due to intimate contact between infected and uninfected flocks in extensive production systems [33]. A higher rate of seropositive has previously been observed in extensive production systems in Ethiopia [25] and Uganda [34] and in pastoral grazing systems [35].

**Table-5:** Farm level risk factors for brucellosis seropositivity in goats.

Criteria	Risk factors	Number of samples (498)	Seropositives (29)	OR and inverse OR^ѱ^ (95% CI)	p-value
Area	Rural	433 (86.95)	25 (86.210)	0.94 (0.32-2.78)	0.908
	Peri urban	65 (13.05)	4 (13.79)	1.07 (0.36-3.16)	
Number of animals in the farm	Small farm	43 (8.63)	8 (27.59)	4.03 (1.68-9.64)	0.001***
	Medium Farm	246 (49.40)	19 (65.52)	1.95 (0.89-4.27)	0.092*
	Large Farm	209 (41.97)	2 (6.90)	0.10 (0.02-0.44)	0.0001***
Method of rearing	Extensive	15 (3.01)	3 (10.34)	3.72 (1.01-13.65)	0.034*
	Semi intensive	483 (96.99)	26 (89.65)	0.27 (0.07-0.99)	
Mode of procurement of animals	Livestock fair	28 (5.62)	5 (17.24)	3.50 (1.24-9.86)	0.012**
	Own raised	470 (94.38)	24 (89.66)	0.29 (0.10-0.81)	
Disposal of aborted materials/foetus	Open discard	290 (58.23)	10 (34.48)	0.38 (0.17-0.83)	0.012**
	Disposed in water bodies	208 (41.77)	19 (65.52)	2.65 (1.21-5.81)	
Separate shed	Yes	209 (41.97)	2 (6.90)	0.10 (0.02-0.44)	0.001***
	No	289 (58.03)	27 (93.10)	9.76 (2.30-41.51)	
Dogs in the farm	Yes	289 (58.03)	27 (93.10)	9.76 (2.30-41.51)	0.001***
	No	209 (41.97)	2 (6.90)	0.10 (0.02-0.43)	
Frequency of Veterinary services obtained	Weekly	289 (58.03)	27 (93.10)	9.76 (2.30-41.51)	0.001***
	Monthly	209 (41.97)	2 (6.90)	0.10 (0.02-0.43)	
Brucellosis Awareness	Yes	93 (18.67)	0 (0.00)	NC	0.001**
	No	209 (41.97)	2 (6.90)	0.10 (0.02-0.43)	

CI=Confidence interval, OR=Odds ratio, NC=Not calculated.

*** p<0.001,

** p<0.01,

* p<0.1

One of the important risks for brucellosis transmission which emerged in the study is the purchase of animals from livestock fairs, a practice which has led to significantly higher OR in goats (OR 3.50, CI 95% 1.2-9.8) than in sheep (OR 1.70, CI 95% 1.0-2.7) (Table-3). Increased disease prevalence due to frequent purchase of animals with an unknown history of brucellosis into the farms has also been similarly reported in Mexico [36] and Malaysia [37]. The other farm-level brucellosis risk observed was the disposal of aborted material in water bodies, which produced significantly higher odds (p<0.01) for both sheep and goats (OR 1.71 and 2.65, respectively). It is a religious belief in some communities the fetuses and placentae should be thrown in water bodies. Furthermore, in some areas, aborted materials are disposed of in the open, where they tend to remain in and around the farm premises for days, which may hasten the acquisition of infection. In the majority of the flocks visited, the animals were huddled together in a single shed, and there was no separation of young, pregnant, or sick animals. Lack of separation at the farms produced the highest odds for goats (OR 9.76, 95% 2.3-41.5) with a significant association to brucellosis (p<0.001). High seropositivity in animals was correlated with exposure to contaminated material, poor management practices, and overcrowding [38,39]. Brucellosis-infected animals act as a source of infection to other animals and hence, it is advisable to separate the animals according to their physiological and health status to prevent the direct transmission of diseases in the farm environment [40].

The presence of dogs on the farms produced a significant association (p<0.001) to brucellosis, with higher odds in goats (OR 9.76, 95% 2.3-41.5) than in sheep (OR 2.89, 95% 1.7-4.7). Dogs always lead or guard sheep and goat flocks, and sometimes enter a farm carrying infected material, or eat the aborted fetuses. Dogs infected with *B. abortus*, in turn, infect cattle [41], and poor biosecurity measures such as lack of control of visitors and stray animals increase the prevalence of brucellosis [42]. The majority of the farms visited were not fenced to prevent the entry of dogs.

Farms in which veterinary consultation occurred once per week showed higher seroprevalence (p<0.001), a finding which did not agree with ­previous reports, which found higher brucellosis seropositivity in farms inaccessible to veterinary services [43]. Abortion and infertility problems should always be investigated by veterinarians to minimize disease transmission. Goat farmers tended to know more about brucellosis than sheep farmers (p<0.001). The education and awareness of the farmer are important for the effectiveness of brucellosis control, as only a few farmers had the habit of consuming raw goat milk for health benefits due to lack of awareness about the potential risks. The inclusion criteria, which only accepted sheep and goat farms, as well as the need for the consent of the farmers, resulted in a limited number of farms being included in the study. The strata specific analysis of ORs showed that age and sex were confounders. In the logistic regression model, abortion (OR 10.8, 95% CI 1.2-96.1), rural farms (OR 8.5, 95% CI 3.6-20.0), and absence of separate sheds in the farms (OR 1.9, 95% CI 1.-3.5) were found to be the significant risk factors for ovine brucellosis (Table-6).

**Table-6:** Multivariable analysis of risk factors for sheep and goat brucellosis in organized flocks.

Species	Factors	Clinical condition	OR (95% CI)	p-value
Sheep	Clinical history/condition	Abortion	10.84 (1.22-96.12)	0.03*
	Area	Rural	8.53 (3.64-20.0)	0.001***
	Separate shed	No shed	1.94 (1.1-3.5)	0.03*
	Constant	−3.925	0.02	0.001***
Goat	Clinical history/condition	No clinical signs	0.05 (0.004-0.595)	0.02*
	Constant	−2.303		0.03*

CI=Confidence interval, OR=Odds ratio, Sheep model: Hosmer-Lemeshow test=2.07, P=0.722, Goat model: Hosmer-Lemeshow test=1.656, P=0.948,

*** p<0.01,

**p<0.01,

* p<0.05

## Conclusion

We identified multiple animal and management risk factors associated with brucellosis in small ruminant farms in the Karnataka state, in the Southern Province of India. The test and slaughter policy is cost-intensive, and therefore in the absence of vaccination, management measures need to be developed to address the risks contributing to the reemergence of brucellosis. The essential management measures include defining the type of rearing, improving husbandry practices, raising farmers’ perceptions of brucellosis, and prioritization of brucellosis control by the government. A package of practices for risk-based control measures to tackle some of the identified risk factors, aimed at veterinarians and farmers, will aid in minimizing brucellosis in the absence of a vaccination policy for small ruminants in India.

## Authors’ Contributions

RS: Conceived and designed the study, carried out the experiment, and drafted the manuscript. TK and NM: Assisted in carrying out the experiment. MN, CY, ORVK, SSa, and KN: Assisted in sample collection and carried out the experiment. ORVK, SS, TK, and KN: Analyzed the data. HR and BRS: Provided guidance and support to carry out the experiments. All authors read and approved the final manuscript.
